# Identification of ER Proteins Involved in the Functional Organisation of the Early Secretory Pathway in *Drosophila* Cells by a Targeted RNAi Screen

**DOI:** 10.1371/journal.pone.0017173

**Published:** 2011-02-23

**Authors:** Vangelis Kondylis, Yang Tang, Florian Fuchs, Michael Boutros, Catherine Rabouille

**Affiliations:** 1 Cell microscopy Centre, Department of Cell Biology, UMC Utrecht, The Netherlands; 2 German Cancer Research Center (DKFZ) and University of Heidelberg, Division Signaling and Functional Genomics, Heidelberg, Germany; Stockholm University, Sweden

## Abstract

**Background:**

In *Drosophila,* the early secretory apparatus comprises discrete paired Golgi stacks in close proximity to exit sites from the endoplasmic reticulum (tER sites), thus forming tER-Golgi units. Although many components involved in secretion have been identified, the structural components sustaining its organisation are less known. Here we set out to identify novel ER resident proteins involved in the of tER-Golgi unit organisation.

**Results:**

To do so, we designed a novel screening strategy combining a bioinformatics pre-selection with an RNAi screen. We first selected 156 proteins exhibiting known or related ER retention/retrieval signals from a list of proteins predicted to have a signal sequence. We then performed a microscopy-based primary and confirmation RNAi screen in *Drosophila* S2 cells directly scoring the organisation of the tER-Golgi units. We identified 49 hits, most of which leading to an increased number of smaller tER-Golgi units (MG for “more and smaller Golgi”) upon depletion. 16 of them were validated and characterised, showing that this phenotype was not due to an inhibition in secretion, a block in G2, or ER stress. Interestingly, the MG phenotype was often accompanied by an increase in the cell volume. Out of 6 proteins, 4 were localised to the ER.

**Conclusions:**

This work has identified novel proteins involved in the organisation of the *Drosophila* early secretory pathway. It contributes to the effort of assigning protein functions to gene annotation in the secretory pathway, and analysis of the MG hits revealed an enrichment of ER proteins. These results suggest a link between ER localisation, aspects of cell metabolism and tER-Golgi structural organisation.

## Introduction

The endoplasmic reticulum (ER) is a very large organelle comprising many subdomains including the rough ER [1], where proteins that need to be secreted to the extracellular medium and most of the transmembrane proteins are synthesised, before being packaged into budding COPII vesicles at the ER exit sites (ERES), also called tER sites, and transported to the ER-Golgi intermediate compartment and the Golgi apparatus. These organelles form the early secretory pathway and during the past decade, we have established that *Drosophila* tissue culture S2 cells are a good model to study its organisation [2].

The *Drosophila* early secretory pathway consists of tER sites closely associated to individual Golgi stacks forming what we, and others, have called tER-Golgi units. In S2 cells, the number of the tER-Golgi units is fairly constant. The molecular principles underlying the organisation of the early secretory pathway are largely conserved between mammals and *Drosophila*, with the exception that in mammalian cells the Golgi stacks are linked into a single-copy organelle, forming the Golgi ribbon.

This morphological similarity extends to a conserved functional organisation and this has been recently exploited by the use of S2 cells in two genome-wide RNAi screens seeking for novel proteins required for the constitutive secretion of soluble reporter proteins [3], [4]. The first one identified a number of TANGO genes [3], the best characterised being Tango1 that mediates collagen loading at ER exit sites [5]. The second identified two novel genes, Grysum and Kish that localize to the Golgi and affect protein secretion upon depletion [4]. These two screens relied on the use of reporters comprising a signal sequence fused to HRP and luciferase, respectively, without direct visualisation of the secretory pathway, at least in the primary screens.

Our previous experience has shown that alterations in the organisation of tER-Golgi units are not necessarily coupled to an inhibition in anterograde transport [6]–[8] and that ER resident proteins can have an impact on this organisation. The latter has been exemplified by the depletion of *Drosophila* CPE synthase (dSMSr), an ER enzyme controlling ceramide homeostasis, which led to a disruption in the number and organisation of tER-Golgi units [9].

**Figure 1:**
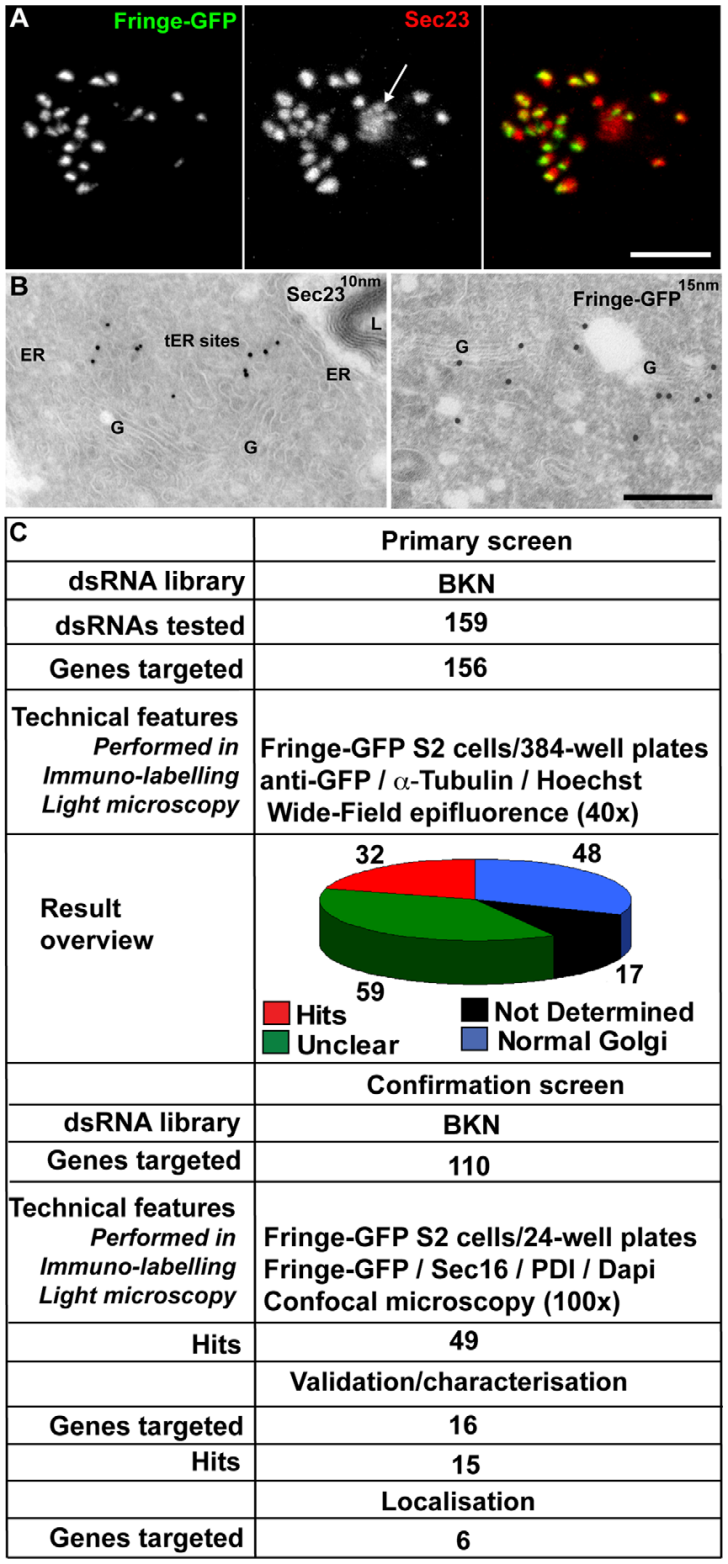
Overview of the RNAi screen. (**A–B**) Organisation of tER-Golgi units in *Drosophila* S2 cells as visualised by IF (A; Fringe-GFP and Sec23 are used as markers for the Golgi stacks and tER sites, respectively), or IEM (B; Sec23 associated with 10 nm gold particles at tER sites and Fringe-GFP with 15 nm on the Golgi stack). tER-Golgi unit organisation was used as readout for the microscopy-based primary and confirmation screens. Arrow in A points at a background nuclear spot of Sec23 antibody. ER, Endoplasmic Reticulum; G, Golgi stacks; L, Lysosome. Scale bars: 5 µm (A) and 200 nm (B). (**C**) Synopsis of primary and confirmation screens, the validation and localisation of a subset of hits (including the number of dsRNAs tested, the dsRNA library used, the readout and type of microscopy used and the number of identified hits).

To investigate the extent that ER-based proteins affect the organisation of *Drosophila* tER-Golgi units, we designed a double screening strategy based on a bioinformatics pre-selection combined to a morphological RNAi screen. We first selected 156 proteins exhibiting known or related ER retention/retrieval signals from a list of about 2500 proteins predicted to have a signal sequence, many of them uncharacterised. The role of these proteins was tested by a microscopy-based RNAi screen in S2 cells, and our approach consisted of 3 phases (see **Figure 1C**): The first was a primary screen designed to detect changes in the pattern of the inducible GFP-tagged Golgi resident enzyme Fringe. The second was a secondary confirmation screen, where genes that led to a clear or ambiguous Golgi phenotype during the primary screen were re-tested using confocal microscopy visualisation of both Fringe-GFP and the tER site marker Sec16, leading to the identification of 49 positive hits. Third, the depletion phenotype of 16 of these hits was further characterised along with the localisation and overexpression phenotype of 6 of them. Interestingly, the disorganisation of the tER-Golgi units (mostly representing an increase in their number) was also accompanied by an increase in the cell volume that we have investigated. Altogether, we find a correlation between the localisation of proteins to the membrane of the early secretory pathway, an effect on the organisation of tER-Golgi units upon their depletion, and an increased cell size. In contrast, no strict correlation was found with anterograde transport, cell cycle progression, lipid biogenesis, TOR activation or ER stress induction.

## Results

### Selection of genes for RNAi depletion

To select for potential ER proteins, we screened a list of about 2500 proteins up to 500 amino acids containing an annotated or predicted signal sequence [10] (provided by Dr. Geert Baggerman, Catholic University Leuven, Belgium) and short-listed those that contain potential ER localisation/retrieval signals at their C-terminus, such as classical ER retrieval motifs. K(x)Kxx is found on ER transmembrane proteins and is important for their COPI-mediated retrograde transport [11], [12]. KDEL or equivalent motifs are responsible for the retention of ER soluble proteins [13], [14]. A double phenylalanine (FF) is shown to mediate ER export through binding to COPII coated vesicles [15] and is present in ERGIC53 [16] and some members of the p24 family of cargo receptors [17], [18]. In addition, proteins with a C-terminus containing two or more lysines were also selected and represent about 50% of the selected proteins. 156 genes encoding potential ER proteins were chosen (**Table S1**) and a gene ontology analysis using DAVID bioinformatics resources tools (version 6.7; http://david.abcc.ncifcrf.gov/) [19] confirmed the enrichment for ER proteins.

### Primary and secondary RNAi screens

#### Design/readout

**Figure 2:**
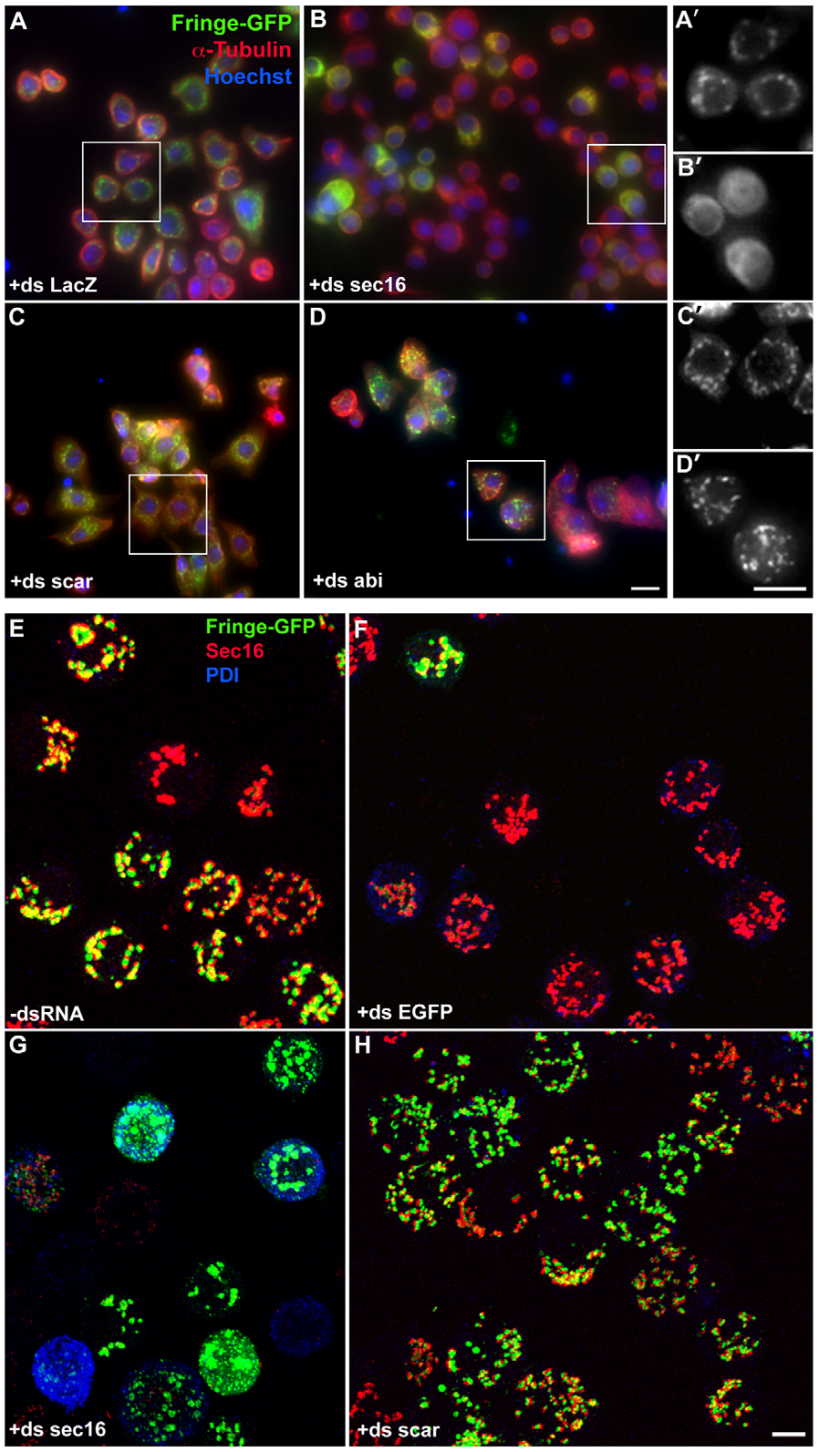
Positive and negative control RNAi depletions in primary and confirmation screens. (**A–D**) Visualisation of Fringe-GFP (inducible Golgi enzyme), microtubule network (α-Tubulin) and DNA (Hoechst) upon different RNAi depletions by wide-field microscopy. Scale bars: 10 µm. (**A'–D'**) Higher magnification of the cells marked by the white squares. Fringe-GFP pattern is shown. As expected, Sec16 depletion resulted in more diffused Fringe-GFP pattern (B', [21]) whereas Scar or Abi depletion led to an increased number of Fringe-GFP spots (C'–D', [8]) compared to mock-depleted cells (A'). Note that the Golgi phenotype can be sometimes difficult to see due to the increased out of focus light. Scale bars: 10 µm. (**E–H**) Visualisation of tER (Sec16)-Golgi (Fringe-GFP) units upon different RNAi depletions by confocal microscopy. ER state was visualised by PDI labelling. Note that Fringe-GFP expression is downregulated in nearly all cells treated with dsRNA against EGFP (F). Sec16 RNAi led to increased diffusion and fragmentation of Fringe-GFP signal (G), while Scar depletion led to a significant increase tER-Golgi unit number coupled to a decrease in size (H). Pictures represent 2D projections of confocal sections. Scale bars: 5 µm.

The primary screen was performed in a stably transfected S2 cell line expressing Fringe-GFP upon induction of a metallothionein promoter (see Materials and Methods). Fringe-GFP is a transmembrane O-linked fucosyl Golgi-resident transferase that is synthesised in rough ER and transported to the Golgi stacks through the ER exit sites [8], [20] (**Figure 1A, 1B**). After incubation with dsRNAs targeting the selected putative ER proteins for 5 days in 384-well plates, Fringe-GFP expression was induced and its localisation was used as readout for the Golgi organisation (**Figure 1C**). This assay allowed us to test both a block in anterograde transport (Fringe retained in the ER) and an effect on the Golgi organisation (Golgi number, size etc). Fixation was followed by α-Tubulin and Hoechst staining to visualize cellular shape and chromosome state/nuclei, respectively. Visualisation was performed using a widefield microscope (**Figure 1C**; see Materials and Methods; **Figure 2A–D**).

In the confirmation screen, we visualised both the Golgi membrane and tER sites using Fringe-GFP and Sec16, respectively (**Figure 2E–H**). Sec16 is an early determinant of tER site biogenesis and exhibits a localization that is undistinguishable from that of COPII coat component Sec23 [21]; therefore, it was used as a tER site marker in all our subsequent experiments. In addition, labelling for protein disulfide isomerase (PDI) was used as an ER marker (**Figures 1C** **, and** **Figure 2E–H**) and Dapi was used to visualize the nuclei (not shown).

#### Positive and negative controls

In both primary and secondary RNAi screens, we performed a number of control depletions to monitor the procedure, as well as the effects on the tER-Golgi organisation. First, in each experiment, DIAP1, an important regulator of apoptosis, and therefore of cell viability [22] was depleted leading to a significant decrease in the cell number [23] (**not shown**). EGFP RNAi was also systematically depleted leading to a significantly reduced expression of Fringe-GFP in more than 90% of the cells (**Figure 2F**). The effect of both control depletions could be easily visualised with an inverted widefield microscope before the rest of the samples were processed for immunofluorescence. Second, mock (no dsRNA added; **Figure 2E**) and LacZ depletions (**Figure 2A,A** **'**) served as negative controls and were used as reference phenotypes for comparison with the selected proteins that were targeted for depletion. Third, depletions of Sec16, Scar/WAVE and Abi were used as positive controls for alterations in Golgi organisation. As previously shown, Scar/WAVE (**Figure 2C,C',H**) and Abi (**Figure 2D,D** **'**) depletion leads to a near doubling in the number of the tER-Golgi units in S2 cells through modulation of actin cytoskeleton [8]. In contrast, Sec16 depletion leads to disorganisation of tER sites and consequentially an inhibition of ER to Golgi transport [21]. Accordingly, Fringe-GFP exhibited a significantly more diffused pattern indicating its entrapment in the ER and this was accompanied by a reduction in the number of Fringe-GFP-positive membranes (**Figure 2B,B',G**). Some depleted cells still displayed Fringe-GFP structures similar to the control cells, but these structures appear mostly (semi) circular and represent ER rather than Golgi membrane, as revealed by EM examination (**not shown**). Finally, a number of cells also showed an increase in PDI levels (**Figure 2G**), probably reflecting the ER accumulation of cargo proteins leading to increased unfolded protein response.

#### Primary screen and hit confirmation

159 dsRNAs (targeting 156 different genes encoding potential ER proteins) were tested for their role in Golgi organisation using BKN amplicons (BKN library [24]; **Figure 1C** **and Table S1**). However, due to the high percentage of dsRNAs exhibiting an unclear phenotype or for which no data were obtained (for definitions see Materials and Methods and **Table S1**; **Figure 1C**), we re-tested all dsRNAs (110), except for those that had convincingly no effect on Golgi organisation in the primary screen. In this secondary/confirmation screen, we used the same dsRNAs but vizualised the samples by confocal microscopy, which is more appropriate to better describe the observed phenotypes (see Materials and Methods) (**Table S2**).

**Figure 3:**
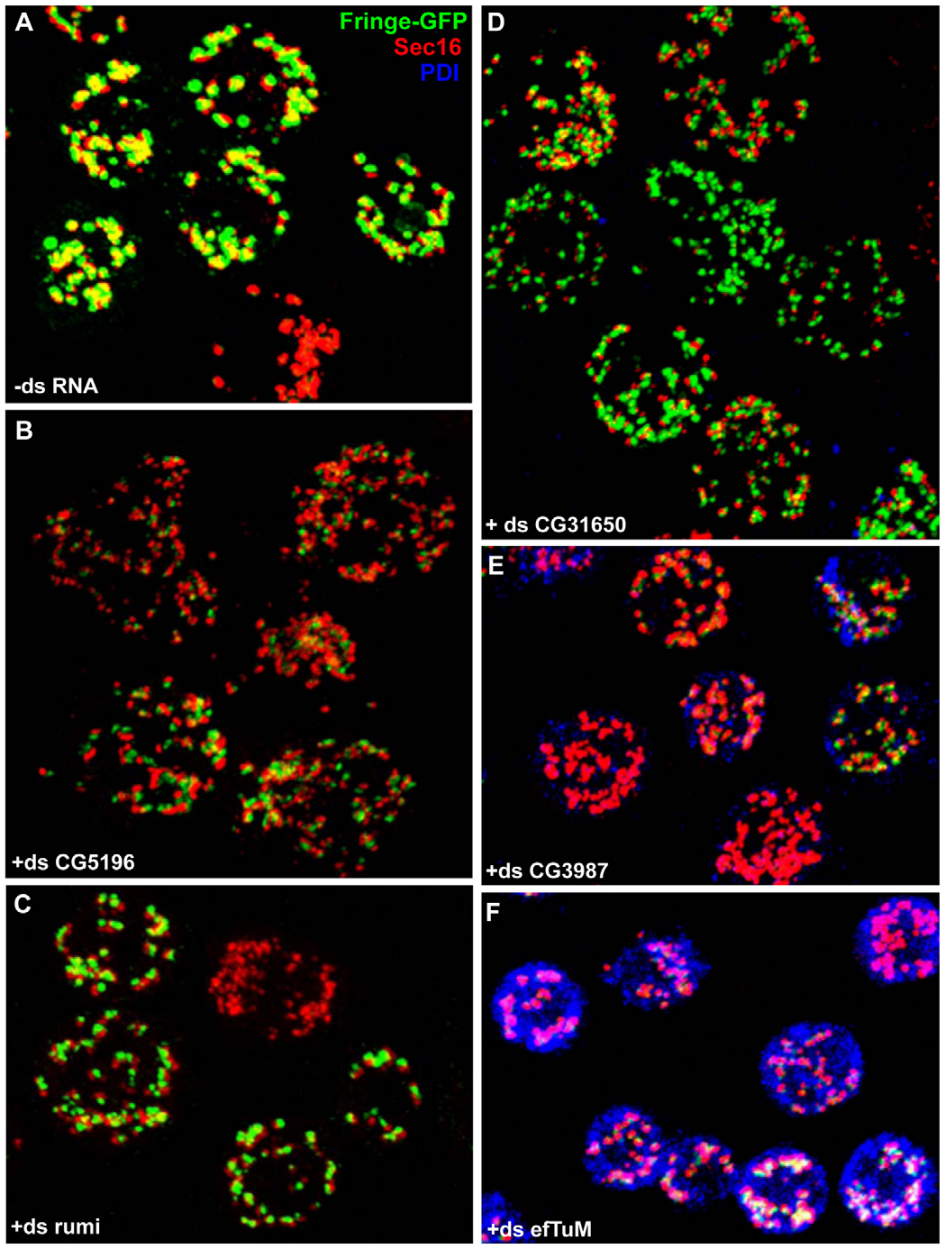
Examples of different phenotypic groups from the confirmation screen. Visualisation of tER-Golgi units (Sec16 and Fringe-GFP, respectively) and the ER luminal marker PDI upon different RNAi depletions by confocal microscopy. (**A**) Visualisation of the typical pattern of tER-Golgi units in mock treated cells (-dsRNA). (**B–D**) Visualisation of the strong MG phenotype (MG+++, ds CG5196, ds CG31650) and moderate (MG++, ds rumi). (**E–F**) Illustration of the decreased Fringe-GFP synthesis and increased PDI staining upon CG3984 and efTuM depletion whereas in the control cells PDI level is very low. The pictures represent 2D projections of confocal sections and some of the panels are composite of images. Scale bar: 5 µm.

In the confirmation screen, 49 out of the 110 dsRNAs tested led to significant changes in the tER-Golgi unit organisation (**Figure 1** **, Tables S1 and S2**). The most commonly observed phenotype (41 out of 49) was an increase in the number of tER-Golgi units in a significant percentage of cells (**Figure 3**, **Table 1**), sometimes accompanied by more diffused GFP staining (haze) or increased levels of PDI (**Table S2**). This phenotype is referred to throughout the text, tables and figure legends as MG for “more and smaller Golgi spots”, and was quantified for 4 candidates (**Table 1**). The hits displaying this phenotype were classified in 3 groups (strong MG+++, moderate MG++ and weak MG+) depending on the phenotype penetrance (**Table S2**). Strikingly, the penetrance of the MG phenotype strongly correlated with an increase in the percentage of large-sized cells (larger cell diameter) in most cases (see below). Gene functional annotation analysis of these hits using DAVID bioinformatics resources [19] has revealed interesting functional clustering (see discussion).

**Table 1:** Quantification of the number and size of Golgi spots and of the cell volume.

	Average number of Fringe-GFP spots*	Average size of Fringe-GFP spots* (µm^2^)	Cell volume(µm^3^)	Number of Fringe-GFP spots*/10 µm^3^
**-ds RNA**	21.6±7	1.1±0.7	381±31	0.57±0.18
**+ds CG10664 (MG+++)**	45±15	0.75±0.5	523±60	0.86±0.28
**+ds CG5196 (MG+++)**	41±14	0.66±0.34	678±53	0.60±0.20
**+ds CG16817** **(MG++)**	36.0±10	0.79±0.4	623±41	0.57±0.16
**+ds CG31650** **(MG++)**	34.6±9	0.75±0.5	838±78	0.41±0.10

*The Fringe-GFP spots represent the Golgi [8].

Among the other phenotypic groups, there was one comprising 4 genes that led to a relatively low Fringe-GFP signal in many cells, small-sized and seemingly fragmented tER-Golgi units, and higher levels of PDI (**Figure 3E–F** **; Table S2;**). This suggested that the depletion of these proteins might have partly affected protein synthesis or transport efficiency along the early secretory pathway. Finally, a third group comprised genes whose deletions led to a tER-Golgi unit aggregation.

### Hit characterisation

#### Cell cycle and anterograde transport

**Figure 4:**
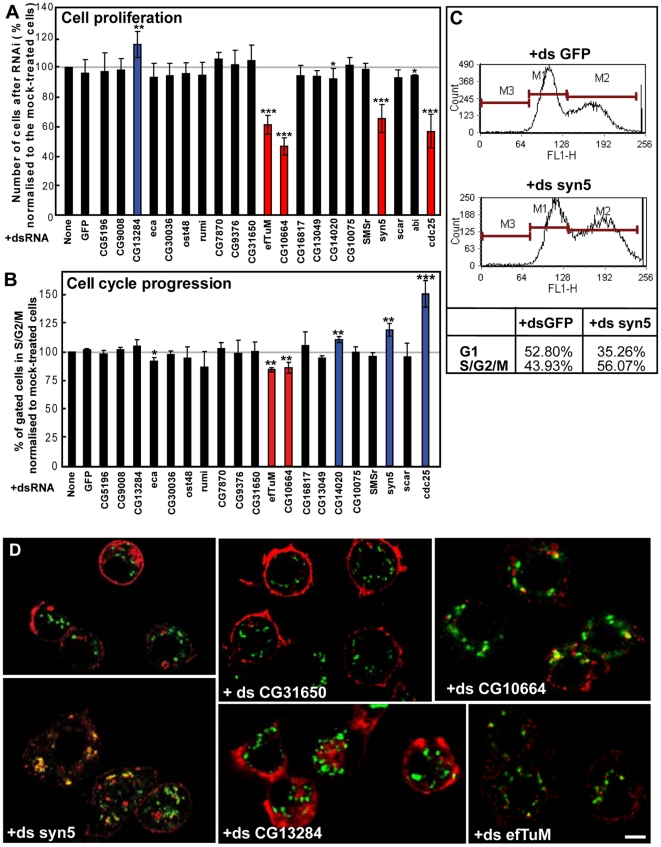
Effect of RNAi depletion of selected hits on cell proliferation, cell cycle progression and anterograde transport. (**A**) Number of S2 cells after 5-day incubation with the indicated dsRNAs expressed as percentage relative to the number of mock-treated cells. Red and blue columns indicate genes whose depletion leads to significant decrease or increase in cell proliferation, respectively. Bars represent SD from at least 3 independent experiments. Conditions with p<0.01, 0.01<p<0.05 and 0.05<p<0.10 are indicated with triple, double and single asterisk, respectively. (**B–C**) Cell cycle distribution of live S2 cells after 5-day incubation with the indicated dsRNAs determined by staining their DNA content. The population of G1 (M1), S/G2/M (M2) or sub-G1 (M3) cells in each condition was quantified by FACS analysis (C). The percentage of gated cells in S/G2/M phase (4N) normalised to the respective value of mock-treated cells, which was considered as 100% (B). Red and blue columns indicate genes whose depletion leads to a significant decrease or increase in the percentage of cells in S/G2/M phase, respectively. Bars represent SD from at least 2 independent experiments. P-values are indicated as in A. Examples of cell cycle distribution and the associated quantification are shown in C. (**D**) Estimation of the efficiency of anterograde transport of Delta S2 cells incubated for 5 days with the indicated dsRNAs, followed by 1-hour induction of Delta with CuSO_4_ and 75 min chase to allow its transport to plasma membrane. Fixed cells were labeled for Delta and dGMAP (cis-Golgi marker). As a positive control, Syntaxin5 (+ds syn5) depletion blocks Delta at the early secretory pathway in most cells (see also [6]. Note that depletion of mitochondrial enzyme EfTuM and CG10664 leads to a significant decrease of Delta synthesis and transport. Scale bars: 5 µm.

Since the MG phenotype was the most frequently observed one in our primary and confirmation screens, we set out to investigate whether there is a common etiology/mechanism underlying it. It has been previously reported that the number of tER-Golgi units increases in G2 phase both in mammalian and *Drosophila* S2 cells [8], [25]. Indeed, experimental block of *Drosophila* S2 cells in G2 phase by depletion of cdc25/String, a key phosphatase for G2/M transition and mitotic entry, leads to accumulation of cells with MG phenotype ([8] and not shown). To assess whether depleted cells with MG phenotype are arrested in G2, we examined cell proliferation (**Figure 4A**) and cell cycle distribution (**Figure 4B,C**) of 16 selected hits after 5-day RNAi depletion. In most cases, cell proliferation was not significantly affected except for two proteins with mitochondria-associated functions (CG10664 and EfTuM; see below). Depletion of these two proteins reduced cell growth to a similar extent as Cdc25 and Syntaxin5 RNAi (**Table 2** **and** **Figure 4A**). Conversely, CG13284 depletion led to a small but significant increase in cell proliferation.

**Table 2:** Characterisation of selected hits.

Gene targeted	tER-Golgi phenotype	Cell proliferation	Cell cycle	Delta transport	Average cell diameter	Lipid droplets	TORactivation
***No dsRNA***	Normal	ok	ok	ok	100	ok	**no**
***GFP***	Normal	ok	ok	ok	99.9±0.2	ok	**no**
**CG5196^#^**	MG+++	ok	ok	mostly ok	108.5±7.5*	**↓**	**no**
**CG10664^#^**	MG+++	**↓↓**	**↑G1**	**↓synthesis/transport**	106.1±2.0*	ok	**no**
**CG13284^#^**	MG++; Aggr?	**↑**	ok	ok	106.3±1.7*	ok	**no**
**CG31650^#^**	MG++	ok	ok	ok	104.1±2.3*	**↓**	**no**
**CG16817^#^**	MG++	ok	ok	ok	103.8±0.9*	**↑**	**no**
**CG13049^#^**	MG++	ok	ok	ok	102.8±0.2*	ok	**no**
**CG9008**	MG+++	ok	ok	ok	104.2±2.1*	**↓↓**	
**efTuM**	MG++/+++	**↓↓**	**↑G1**	**↓synthesis/transport**	102.2±2.3	ok	**no**
**CG10075**	MG++	ok	ok	ok	98.6±1.6	**↑**	
**CG7870**	MG++	ok	ok	ok	99.5±0.6	ok	
**ost48**	MG++	ok	ok	mostly ok	102.0±1.5*	**↓↓**	
**rumi**	MG++	ok	ok	ok	103.3±2.2	ok	**no**
**CG30036**	MG+/++	ok	ok	ok	102.5±0.3*	ok	
**eca**	smaller size tER-Golgi	ok	ok	mostly ok	99.3±1.7	**↓↓**	**no**
**CG14020**	MG+/++; Aggr	ok	**↑S/G2/M**	ok	103.8±1.5*	ok	
**CG9376**	Normal	ok	ok	ok	97.8±3.6	**↓**	
***SMSr***	MG++	ok	ok	ok	**107.7±2.8**	ok	
***syntaxin5***	Golgi vesiculation	**↓↓**	**↑↑S/G2/M** ‡	**↓transport**	**110.8±1.9**	**↑**	
***scar***	MG+++	ok	ok	ok	**107.1±2.8**	ok	
***abi***	MG+++	ok	nd	ok	nd	nd	
***cdc25***	MG++++	**↓↓**	**↑↑S/G2/M** ‡	ok	**121.6±6.3**	ok	**no**
***Metaphase***	tER-Golgi haze	nd	nd	nd	**132.1±1.7**	nd	

The controls are marked in *italics*. The subcellular localisation of the candidates marked with # was further analysed (in Table 3).

**tER-Golgi phenotype**: **MG**, More and smaller tER-Golgi units. The phenotype ranges from ++++ (strongest) to + (marginal). See also Table S2.

**Cell proliferation**: Double or single arrows indicate statistically significant (p<0.05) or a tendency (0.05<p<0.15) for increase or decrease in cell proliferation compared to mock-treated cells, respectively. See also Figure 4A.

**Cell cycle:** Conditions resulting in a G1 or S/G2/M block are marked by double or single arrows depending on statistical significance.

‡ indicates conditions with increased sub-G1 cell population. See also Figure 4B.

**Average cell diameter** (normalized to mock-treated cells): Values marked in bold indicate hits with p<0.05 and asterisks indicate hits with 0.05<p<0.15.

**Figure 5:**
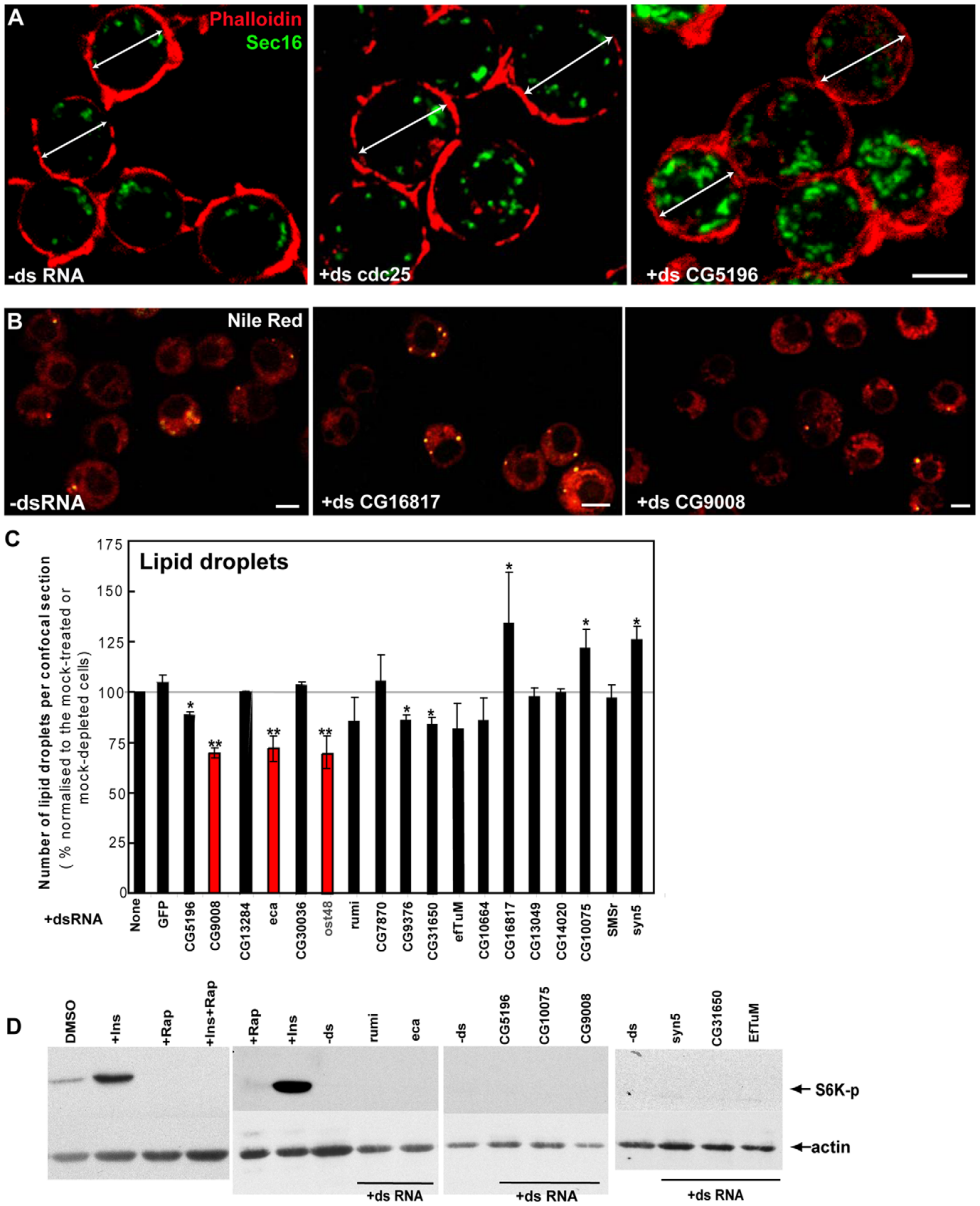
Effect of RNAi depletion of selected hits on lipid droplet formation and TOR pathway activation. (**A**) Visualisation of the cortical actin (phalloidin, red) and Sec16 (green) in non-depleted and cdc25 and CG5196 depleted cells. The white dual arrow indicates the cell diameter. Scale bars: 10 µm. (**B**) Visualisation of the lipid droplets with Nile red (10 min) in S2 cells incubated for 5 days with the indicated dsRNAs. Note the increase or decrease in the number of lipid droplets upon depletion of CG16817 or CG9008, respectively, as compared to the mock-treated cells. Scale bars: 10 µm. (**C**) Number of lipid droplets per equatorial confocal cell section quantified and expressed as percentage normalised to the respective value in mock-treated cells, which was set as 100%. Red and blue columns indicate proteins whose depletion leads to a significant decrease or increase in the number of lipid droplets (marked with double asterisk; p<0.05). Depletion of proteins that led to a tendency for higher or lower number of lipid droplets, which was not statistically significant (0.05<p<0.20) are marked by one asterisk. Bars represent SEM from at least 3 independent experiments. (**D**) Western blot of Phospho-S6K on lysates of S2 cells incubated in DMSO, rapamycin, insulin, insulin+rapamycin and S2 cells incubated for 5 days with the indicated dsRNAs.

**Lipid droplets:** Double or single arrows indicate statistically significant (p<0.05) or a tendency (0.05<p<0.15) for increase or decrease in lipid droplet number compared to mock-treated cells, respectively. See also Figure 5B–C.

**TOR activation:** “no” indicates the protein depletions with no increase in phospho-S6K. See also Figure 5D.

To examine cell cycle distribution, depleted living cells were stained with a DNA-binding dye and FACS-sorted according to their DNA content. As expected by the cell proliferation quantification, cell cycle profiles for most protein depletions were similar to non- or mock-depleted cells. Exceptions were the two mitochondria-related proteins that when depleted led to a 10% increase in G1 cell population, indicating that the cell growth inhibition was due to a G1 arrest/delay (**Figure 4B,C**). This is different from Cdc25 and Syntaxin5 depletion that reduce cell proliferation by arresting cells in G2 or blocking cytokinesis (**Figure 4B,C**). Furthermore, depletion of CG14020, an uncharacterised protein with predicted N-acetylglucosamine sulfotransferase activity, resulted in a slight increase in S/G2/M cell population (**Figure 4B**), suggesting it could be a novel (in)direct regulator of G2/M transition or cytokinesis.

An increase in the number of tER-Golgi units could also result from a fragmentation of the early secretory pathway due to a disruption in anterograde transport, as observed upon depletion of Syntaxin5 (**Figure 4D**) or the class B hits identified by Bard *et al* [3] in their genome-wide screen. To test this, we used a robust assay that monitors the deposition of the transmembrane protein reporter Delta to the plasma membrane [6]. In most cases, anterograde transport was unaffected (**Table 2** **;** **Figure 4D**). Again, the only exceptions were CG10664 and EfTuM, whose depletion inhibited Delta transport to the plasma membrane but also reduced its synthesis (**Figure 4D**). Taken together, these results confirm on a large scale the earlier finding that the disorganisation of the early secretory pathway does not necessarily block anterograde transport and cell proliferation suggesting a great flexibility of the pathway at least in *Drosophila* [2].

Overall, these observations indicate that neither a defect in cell cycle progression nor a block in anterograde protein transport can provide a unifying explanation for the MG phenotype observed in most RNAi depletions.

#### The big cell phenotype

Strikingly, in the primary screen, the MG phenotype penetrance strongly correlated with an increase in the percentage of large-sized cells, a phenotype we called BC for “big cells” (**Figure 3** **and** **5A**). To quantify the BC phenotype, we measured the average cell diameter after depletion of our selected hits (see Materials and Methods) and found a measurable increase in cell diameter (up to 8.5%; values marked by asterisks in **Table 2**). Considering that no obvious cell shape changes were observed upon F-actin staining by phalloidin **(** **Figure 5A** **)** and that the S2 cell shape approximates that of a sphere, this increase in cell diameter could represent an increase in cell volume up to 28%.

Next, we set out to investigate the mechanism leading to the increased size of these cells. First, a block in G2 could lead to increased cell size, as it is the case upon Cdc25 depletion (**Figure 5A** **,** **Table 2**) [8]. As mentioned above, however, none of the selected protein depletion considerably increased the G2 cell population. Second, an increase in the anterograde transport rate or inhibition of endocytosis could increase the cell surface to accommodate a larger cell volume. Nevertheless, none of these two parameters seem to be significantly affected in the BC hits judging from Delta deposition to PM and its subsequent endocytosis (**Table 2** **,** **Figure 4D**).

Third, we investigated the number of lipid droplets as this aspect of lipid metabolism could be affected in the depleted cells. Indeed, increasing evidence suggests that lipid droplets also regulate important aspects of cellular homeostasis, for instance through ribosomal translation, and act as signaling-initiating compartments [26], [27]. Furthermore, lipid droplet biogenesis has been associated with proteins involved in trafficking through the early secretory pathway and changes in its functional organisation might lead to a change in the biogenesis pathway [28]–[30]. In this respect, one of the hits we got in our screen, the putative ER protein CG9008 (MG++), is also part of the lipid droplet proteome [31]. Although addition of oleate in culture medium is often used to study lipid droplet formation and homeostasis [28], [32], we performed our experiments in normal culture medium. In these conditions, mock-treated cells exhibited 0.61±0.21 lipid droplets per confocal equatorial cell section and more than half of the tested hits did not deviate from this number (**Figure 5B–C** **,** **Table 2** **and not shown**). Several depletions, however, significantly decreased (CG9008; eca; Ost48) the number of lipid droplets, whereas others showed a trend toward decrease (see single asterisks in **Figure 5C**) or increase (including CG16817 and Syntaxin5) when compared to control cells. These need to be further investigated but overall, these results, although potentially interesting, cannot provide a common explanation for the BC phenotype for all protein depletions tested.

Finally, we reasoned that the BC phenotype could be due to an activation of TOR upon depletion of a number of hits. TOR is a major regulator of cell growth and metabolism (positively regulating protein and lipid synthesis) that is stimulated by insulin/mitogen/growth factor signaling and molecular sensors of nutrient and energy availability [33], [34]. More specifically, TOR activation positively regulates protein and lipid synthesis, as well as ribosome and mitochondria biogenesis, all important aspects of cellular growth. To test this, we assessed the phosphorylation level of Thr389 of S6K, one of the TORC1 complex targets [35]. Control cells display a small amount of S6K phosphorylation that was greatly enhanced by insulin and completely abolished by rapamycin treatment (**Figure 5D**). However, depletion of none of the hits leading to the BC phenotype stimulated S6K phosphorylation, ruling out the involvement of TOR pathway in the observed phenotype.

As most of the RNAi depletions leading to BC phenotype displayed also strong or moderate MG phenotypes, we assessed whether there was a causal relationship between the increase in the Golgi number and the increase in cell size. To address this, we quantified the number of fluorescently-labeled Golgi and the cell volume upon RNAi depletion of 4 representative candidates using ImageJ, and calculated the ratio of these two parameters (assuming that S2 cells have spherical shape; **Table 1**). We reasoned that if the Golgi number and cell size were causally linked (for instance, if each tER-Golgi unit was associated to a defined volume of cytoplasm), then increasing the cell size or the number of tER-Golgi units would proportionally increase the associated parameter. As expected, the number of Golgi spots and the cell diameter were significantly increased upon depletion of the selected hits when compared to non-depleted cells, and the ratio did not vary significantly in 3 out of the 4 depletions quantified, suggesting that the posed hypothesis could be correct. However, for CG10664-depleted cells, the ratio significantly increases, suggesting additional factors could influence the relationship between the two parameters, leading to a higher increase rate in the number of Golgi compared to the cell size (see discussion).

### Localisation of selected hits

To assess whether proteins scored as hits in our screen have a direct or indirect role in the organisation of the tER-Golgi units and whether there is a correlation between specific localisation and the BC phenotype, we expressed 6 C-terminally V5 tagged hits and examined their cellular localisation as well as any dominant negative effect on the tER-Golgi unit organisation (**Table 3**). The 6 selected hits exhibited a strong or moderate MG and BC phenotype, comprised proteins with different C-terminal motifs (see above) and have not been previously characterized. Information on these hits can be found in (http://flybase.org/).

**Table 3:** Localisation/overexpression of selected candidates.

Gene	tER-Golgi phenotype	Protein functional domains	Localisation	Effect of overexpression	Additional effects
CG5196 (BC)	MG+++	Zinc finger DHHC-type, SH3, Palmitoyl-transferase; KKxx-COOH	**ER**	No	At PM in some cells (leak?)
CG10664 (BC)	MG+++	Cytochrome c oxidase subunit IV; KxKx-COOH	**Mitochondria**	Slight ↓ tER-Golgi number, ↑size (elongated shape), less Sec16 haze	
CG13284-PA CG13284-PB (BC)	MG++; Aggr?	Steroid- dehydrogenase; KxKKxx-COOH	**ER**	No	Karmellae in some cells
CG31650 (BC)	MG++	Multiple EF-hand, Ca^2+^-binding, CREC/reticulocalbin protein family; SDEL-COOH	**ER/tER sites**	No	
CG16817 (BC)	MG++	HSP20-chaperone domain; KKxxx-COOH	**ER** with very thick nuclear envelope staining+nucleus	No	
CG13049	MG++	No known domains; PYFF-COOH	**Golgi**	No	

**Figure 6:**
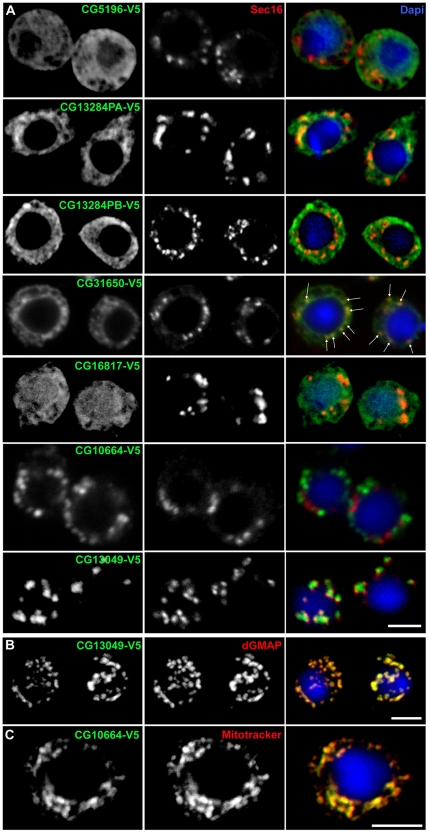
Localisation/overexpression of selected hits. (**A**) Immunofluorescence (IF) localisation of six V5-tagged hits in *Drosophila* S2 cells using an anti-V5 monoclonal antibody (green) and an anti-Sec16 antibody recognizing endogenous Sec16 (red). The merged channels are shown in the third column together with Dapi. Both CG13284 isoforms were expressed and exhibited similar ER localisation. Arrows in CG31650 panel point at puncta of more concentrated staining that partially overlap with Sec16, likely representing tER-Golgi units. (**B**) IF localisation of CG13049-V5 (green) with respect to the Golgi that is labeled with an anti-dGMAP antibody (red). (**C**) IF localisation of CG10664-V5 (green) with respect to mitochondria that are stained with mitotracker (red). Note in B and C the complete overlap between the tagged proteins and Golgi membrane and mitochondria, respectively. Representative confocal sections are shown. Scale bars: 5 µm.

Out of these 6 proteins, only three have classical ER retention motifs and indeed, they were localised to the ER membrane as shown by their reticular fluorescent pattern reminiscent of the ER network in S2 cells and their co-localisation with the ER marker PEMT-GFP (see Material and Methods, **Figure 6** and **Figure S1**, [9]). **CG5196**, one of the strongest depletion MG hit with a BC phenotype, is predicted to have 2 putative isoforms (427 and 395 aa), 4 transmembrane domains (TMD), a cytoplasmic C-terminal KKxx motif (using different Expasy proteomics tools; http://expasy.org/tools/) and contains a zinc finger DHHC and a SH3 domain. It has homology to a mammalian enzyme predicted to be palmitoyl-transferase. The longest of the *Drosophila* isoforms is localised to the ER in S2 cells, though in some cells, the plasma membrane is slightly labeled probably reflecting the leakiness of the retrieval machinery (**Figure 6A** **; Figure S1**). **CG13284**, a moderate MG hit with a BC phenotype, contains a C-terminal KKxKxx and is predicted to be a type 1 transmembrane protein with 2 isoforms (325 and 339 aa). It is proposed to encode the short chain of the mammalian steroid dehydrogenase/reductase similar to the microsomal beta-keto-reductase in *S. cerevisiae* and *P. pastoris*. Both isoforms display a network-like distribution characteristic of the ER morphology in S2 cells (**Figure 6A**), and they form karmellae in some cells. **CG31650**, a moderate MG hit with a BC phenotype, is predicted to be a 342 aa soluble ER multiple EF-hand Ca^2+^-binding protein with a C-terminal SDEL motif. It is homologous to the mammalian reticulocalbins. These are members of CREC protein family (Ca^2+^-binding protein of 45 kDa (Cab45), reticulocalbin, ER Ca^2+^-binding protein of 55 kDa (ERC-55), and calumenin), which localize to different parts of the secretory pathway and are implicated in a variety of pathological conditions [36]. CG31650 localizes to the ER (**Figure 6A** **; Figure S1**) and is enriched in tER-Golgi units in S2 cells (**Figure 6A**, white arrows).

Furthermore, **CG16817**, a moderate MG hit, encodes a 184 aa (1 isoform) soluble protein with an extreme C-terminal KKxxx and contains an HSP20-like chaperone domain that binds to and regulates Hsp90 family chaperones in *Pichia pastoris*. It is also found to localize to a certain extent to the ER (**Figure 6A**) but part of it seems to be confined to the nucleus (**Figure 6A** **; Figure S1**, double arrows). To sort out whether this represented a thickening of the nuclear envelope, co-localisation with PEMT-GFP was performed, but this ER marker did not show this “nuclear” localisation (**Figure S1**). This suggests that CG16817, despite the fact that it has a predicted signal sequence, it is more likely to be a cytosolic protein associated to the cytoplasmic leaflet of the ER with a fraction of it possibly shuttling to the nucleus.

On the other hand, the moderate MG hit **CG13049** is predicted to have 2 isoforms (223 and 181 aa) and 2-6 TMDs (depending of the prediction program used). Both isoforms have a PYFF sequence at their extreme C-terminus and are extremely rich in alanine and proline residues, suggesting they could bind to SH3-containing proteins. Its function is unknown and it is a Drosophila-specific protein that localizes to the Golgi apparatus in S2 cells (**Figure 6A-B**).

Finally, **CG10664**, a strong MG hit with BC phenotype, which appears to inhibit protein synthesis/transport (**Figure 4D**) and G1/S transition (**Figure 4A–B**), exhibits a C-terminal KKxKx motif and is predicted to encode a 182 aa putative *Drosophila* homologue of cytochrome C oxidase subunit IV. As expected, it localizes to mitochondria and its overexpression leads to a slight decrease in the number of tER-Golgi units that appears more elongated and compact (**Figure 6A and C**). This localisation is in agreement with the similar phenotype we observe upon depletion of CG10664 and the mitochondrial elongation factor EfTuM [37].

Taken together, these results show that 4 out of 6 tested proteins localize to the early secretory pathway (ER, tER sites or Golgi), vindicating our method of selection through the C-terminal motif. Although their overexpression did not affect significantly the structural organisation of the tER-Golgi units, their localisation likely justifies the observed effect on tER-Golgi units upon depletion. It suggests, therefore, that these proteins could play a role as structural regulators of the early secretory pathway but without significantly affecting anterograde protein transport. Furthermore, the depletion of these proteins increases the cell size suggesting a correlation between ER localisation and the big cell phenotype (see discussion).

### ER stress

Since a number of the proteins localised at least partially to the ER, and considering the enrichment of our screen targets in annotated ER proteins and the significantly higher hit rate compared to other published RNAi screens (see discussion), the MG phenotype could simply be a consequence of depleting any ER resident protein. However, the depletion of at least 6 *bona fide* ER proteins (p24 protein Baiser (CG11785), ER chaperones Calreticulin (CG9429) and Boca (CG30498), PDI-like enzymes ERp60 (CG8983) and CaBP1 (CG5809) and the signal recognition particle receptor subunit SsRbeta (CG5474)), in the primary and/or the validation screen did not affect the organisation of tER-Golgi units in the majority of S2 cells (**Figure S2; Table S1 and not shown**) in the same conditions (5-day incubation with the corresponding dsRNAs) that led to the MG phenotype when hits were depleted. Although the formal proof that all these proteins are efficiently depleted is lacking, the considerable number of ER proteins with no MG phenotype argues against an unspecific/generalized effect on tER-Golgi organisation upon any ER protein depletion.

**Figure 7:**
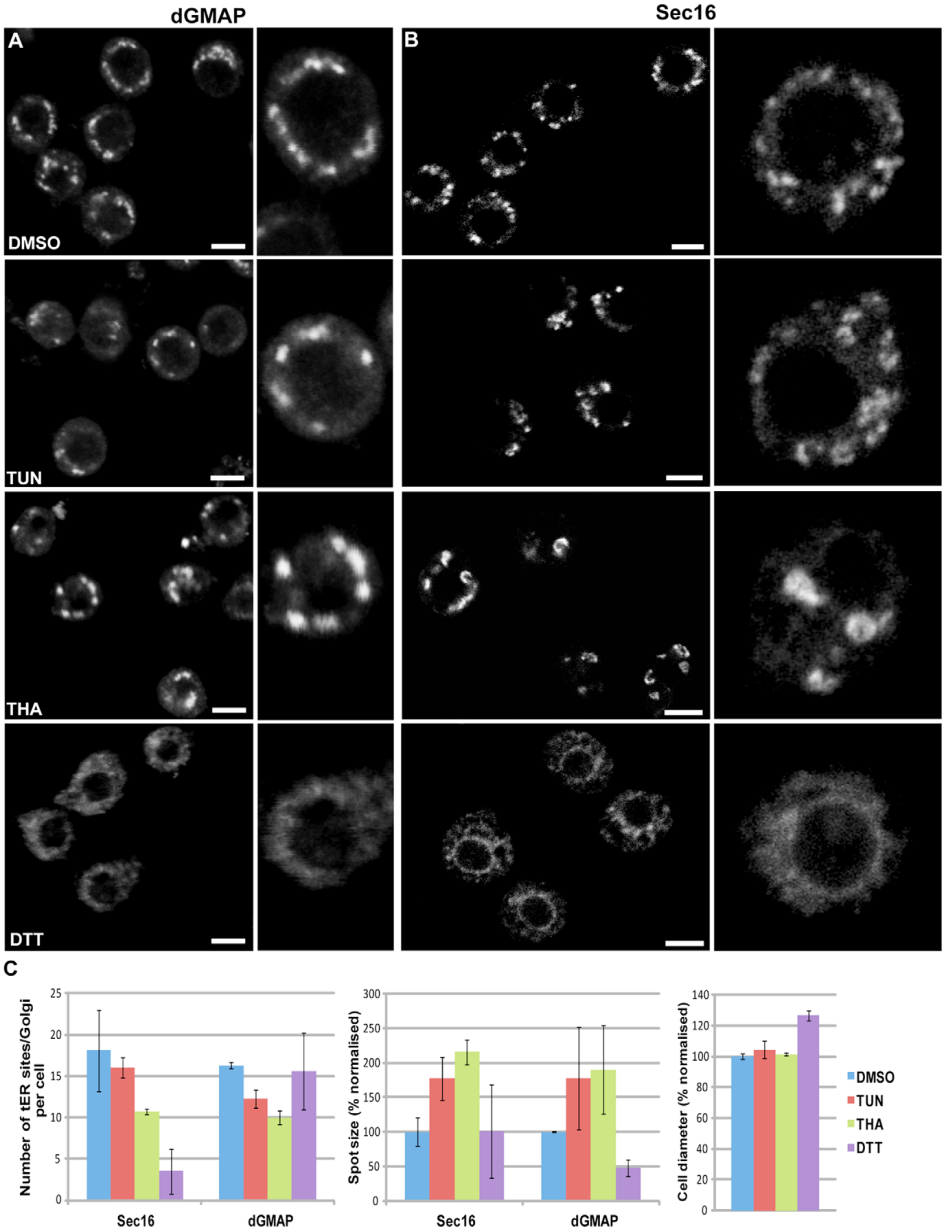
Induction of ER stress does not lead to a MG phenotype. (A–B) S2 cells were treated with vehicle (0.1% DMSO), 10 µg/ml tunicamycin (TUN), 1 µM thapsigargin (THA) and 5 mM DTT for 24 h, fixed and processed for IF. Single labelling for dGMAP (Golgi, A) and Sec16 (tER site, B) was performed. Examples in high magnification are on the left. Note the (semi) circular structures labeled for Sec16 in THA- and to a lesser extent in TUN-treated cells, and the tER-Golgi unit enlargement in these cells compared to the controls. Note also the Golgi fragmentation/dispersal and Sec16 redistribution to the general ER upon DTT treatment. Pictures represent 2D projections of confocal sections. Bars: 5 µm. (C) Quantification of the number of tER (Sec16) and Golgi (dGMAP) spots per cell, their size and the cell diameter upon incubation with the different reagents for 24 h. The size of the fluorescent puncta and cell diameter are expressed as percentages normalized to the values of the control cells that were set as 100%. Note the significant decrease in the number of tER-Golgi units in THA- and TUN-treated cells and the increase in their size. Bars represent ±standard deviation.

Finally, a plausible explanation how the depletion of several ER proteins could lead to the MG phenotype could be through induction of ER stress. To test this hypothesis, we treated S2 cells for up to 24h with tunicamycin, thapsigargin or DTT, all known reagents causing ER stress through different molecular mechanisms [38], [39], and assessed the tER-Golgi unit organisation. Interestingly, these agents seem to modify the organization of the early secretory pathway, but none leads to an MG phenotype. Treatment of S2 cells with DTT at a concentration previously described [39] (for 4–8 h resulted in a pronounced redistribution of Sec16 probably to the general ER (as shown by the labelling of the nuclear envelope), fragmentation/dispersal of the Golgi marker dGMAP, and disruption of the cellular integrity (**Figure 7A,B** **and not shown**). In contrast, treatment for 8 and 24 h with thapsigargin and to a lesser extent with tunicamycin led to an apparent reorganisation of the tER site marker Sec16 into semi-circular and ring-like structures (**Figure 7A**), a decrease in the Golgi number and a slight increase in their cell size (4% larger diameter for tunicamycin-treated cells compared to control cells) (**Figure 7A–C**). The reduced tER-Golgi number did not lead to a decreased cell size, arguing against a strict correlation between cell size and number of tER-Golgi units. Altogether, these results show that ER stress induction leads to different effects on the organisation of the early secretory pathway than those observed in our screen, and provide additional support to the specificity of the MG phenotype.

## Discussion

### New insights into secretory pathway using RNAi screens

The complete sequencing of the human genome has generated a vast amount of information [40] and an obvious task for the future is to discover the biological function of all annotated genes and their role in cell and developmental biological processes. The development of *in vivo* functional assays in model organisms, such as mice and *Drosophila melanogaster*, as well as in tissue culture cells is therefore crucial for this purpose. A revolutionary methodology developed the last decade towards this direction is the set up of genome-wide RNAi screens [41], [42].

One important cell biological question is how intracellular membrane compartments are built and maintained, including these of the early secretory pathway. Over the past three decades, genetic, biochemical and morphological analyses have significantly advanced our understanding of the molecular machineries mediating its organisation and key function in secretion [43]. Critical players have been identified with a role in ER to Golgi and Golgi to plasma membrane trafficking, and some have been specifically implicated in biogenesis/maintenance of its structural architecture.

The function of the exocytic pathway in protein secretion has been recently addressed by two genome-wide RNAi screens in S2 cells using luciferase/HRP-based assays, which are quick and easy to quantify, leading to the identification of some novel regulators of secretion (see introduction; [3], [4], [44]. However, the identification of new proteins involved in its structural organisation is a more challenging task, because it requires microscopy-based assays that are much more laborious. Although automated pattern recognition software that facilitates the unbiased analysis and classification of different phenotypes are being developed [45], [46], application at a genome-wide level is not yet common practice, due to the massive amount of generated data. Instead, less time-consuming, smaller screens targeting a specific functional group of genes (for example, kinases, phosphatases, transmembrane proteins, actin-regulators, druggable proteins) have been performed providing important insight into various cellular processes [47]–[50]. For instance, a recent RNAi screen of mammalian kinases and phosphatases showed the involvement of MAPK/ERK signalling cascade in regulating organisation of early secretory pathway through ERK2-mediated phosphorylation of Sec16 [51].

In the current study, we undertook such a targeted approach by applying two screening strategies to analyse the role of ER resident proteins in the organisation of *Drosophila* tER-Golgi units. We combined bioinformatics and morphology-based RNAi screening, a highly selective approach that is complementary to previous genome-wide screening approaches. First, the selection of the proteins on basis of their C-terminus ER retention/retrieval motifs or similar lysine-containing sequences has proven successful as gene functional annotation analysis confirmed the significant enrichment in ER proteins. Second, an RNAi screen was performed to investigate the role of these proteins in the organisation of the secretory pathway using a light microscopy-based assay (**Figure 1** **and** **8**). Depending on the read-out, the issue with all microscopy screens is to always find an optimal balance between the time required for the data acquisition and the quality of the acquired images. This was challenging in our set-up considering that S2 cells are semi-adherent, much smaller than mammalian cells, and that the investigation of the tER-Golgi organisation requires imaging of nearly the entire cell volume, as the S2 cells are round. Therefore, we analysed the primary screen by quick manual/visual inspection to exclude proteins whose depletion did not have an effect, and invested more time in re-testing the 110 proteins using confocal microscopy, a more appropriate method for the analysis of intracellular organelle organisation.

**Figure 8:**
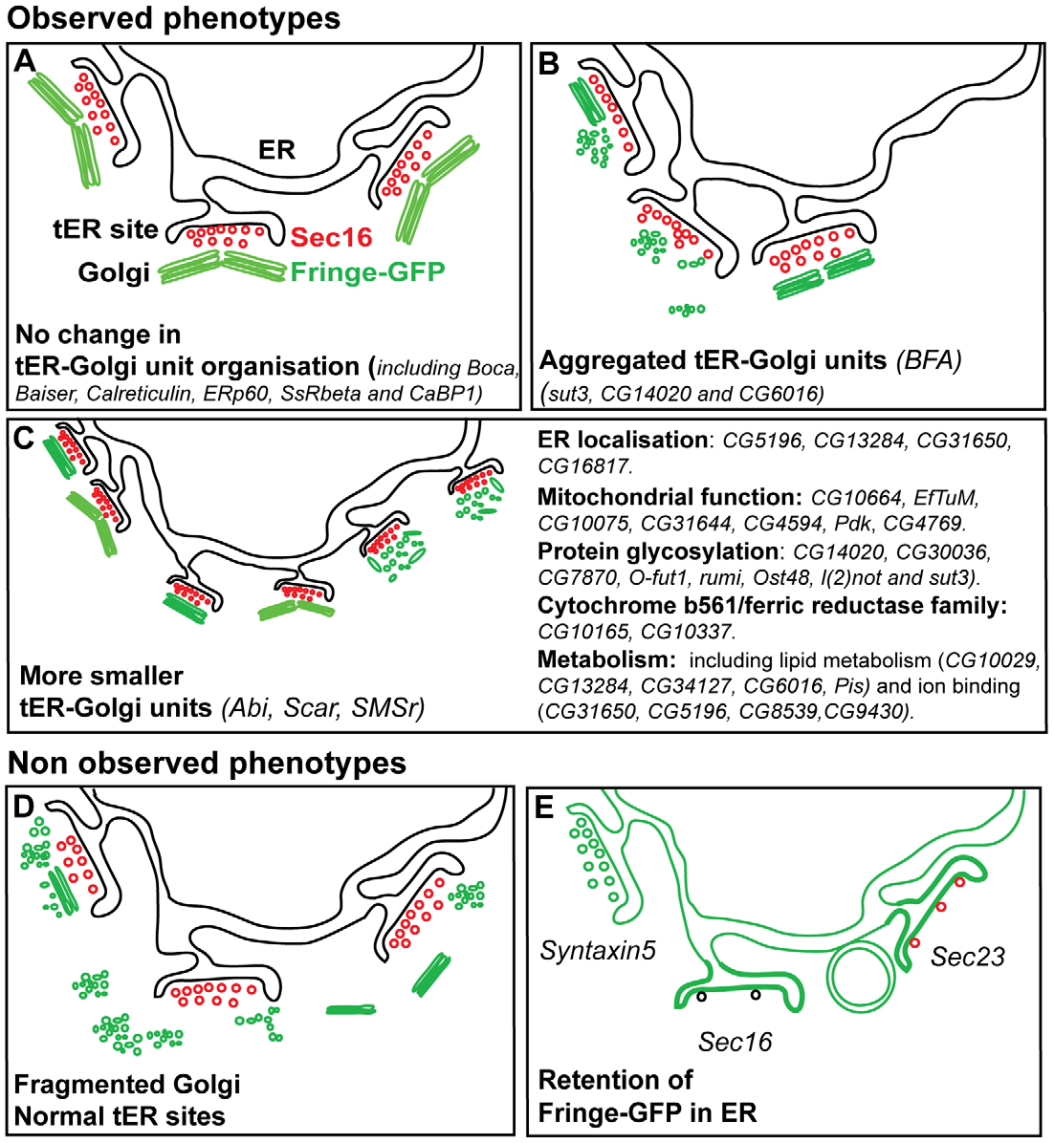
Screen phenotypes. (**A**) No change in the organisation of the tER-Golgi units when compared to non depleted cells. The tER sites are depicted with COPII vesicles decorated by red Sec16, and the Golgi apparatus is represented as a green paired Golgi stacks marked by Fringe-GFP [8]. This phenotype was obtained for 2/3 of the depleted genes. (**B**) Aggregated phenotype: The tER-Golgi units appear aggregated on one side of the cells and seemingly larger. Again, the Golgi apparatus can have retained its wild-type organisation (as in cells treated with BFA, [8]) or be fragmented. This phenotype was observed in 4 out of 49 hits. (**C**) MG phenotype: the tER sites retain their spatial relationship to the Golgi as in non-depleted cells but they appear smaller. However, whether the two compartments have an altered structure is not assessed. The Golgi apparatus can be a smaller paired stack, or a single stack (as in Abi and Scar depletion; [8]) or fragmented into vesicles and tubules (as in SMSr depletion; [9]). This phenotype is observed in 41 out 49 hits. The most prominent functional groups of proteins, whose depletion leads to this phenotype, are listed. (**D**) The Golgi is heavily fragmented, retains or not its spatial relationship with the tER sites that are not affected. This phenotype was not observed. (**E**) Fringe-GFP is retained in the ER and the tER sites (as in Syntaxin5 depletion). It might also accumulate in the tER sites or in circular structures as observed in Sec16 depletion (only few COPII vesicles formed (black), [21]) or depletion of its receptor. Last, any depletion affecting COPII formation (such as Sec23 depletion) could lead to the same phenotype except that the small number of COPII vesicle formed would still be positive for Sec16 (red).

This resulted in the confirmation of 49 hits that exhibited alterations in tER-Golgi organisation, which represents about one third of the proteins tested, many of them novel. This suggests that the morphology of the tER-Golgi units is very dynamic and labile or/and that many proteins can directly or indirectly contribute to it (see below). This high percentage of positive hits is also due to the over-representation in the target list of proteins associated with the ER, which is functionally and structurally related to the tER-Golgi units, therefore vindicating our approach.

### Phenotypic analysis

Our screening assay was designed to detect anterograde transport-independent structural changes of the tER-Golgi units, but also changes that result from an inhibition in ER-Golgi transport. The former phenotype has been exemplified upon the combined depletion of dGRASP/dGM130, which strongly affect the Golgi stack architecture without disrupting protein transport (**Figure 7D**; [2], [6], [7]. This has not been observed, possibly due to the resolution limit of the fluorescence microscopy that does not allow detecting small changes in the Golgi structure. Therefore, proteins whose depletion causes such alterations may have been missed in our screen (**Figure 8A**).

The latter phenotype (inhibition of transport) has been observed in two different experimental conditions. Depletion of a protein, such as Syntaxin5, would lead to a block of Fringe-GFP in the ER and tER sites, the organisation of which would be normal (**Figure 7E**, [6]). A similar ER arrest could be observed upon depletion of proteins that would disperse the tER sites, such as Sec23, the scaffolding protein Sec16 [21], [52]–[55], or its elusive transmembrane ER receptor (**Figure 8E**). In this case, Fringe would also have accumulated at tER sites and in ring-like structures (**Figure 2** **and not shown**) [52]. However, this phenotype was also not observed. As all unclear and non determined primary hits were included in the secondary/confirmation screen, this suggests that either the Sec16 receptor was not included in the list of targeted proteins or that other redundant mechanisms kept Sec16 in place even when the receptor was depleted. Altogether these results are in line with the fact that the hits from our screen do not overlap with those from Bard and colleagues [3] and Wendler and colleagues [4].

An observed but not frequent effect upon depletion of 3 hits (sut3, CG14020 and CG6016) was the aggregation of tER-Golgi units (**Figure 8B**). In addition, 4 hits also exhibited a reduced Fringe-GFP signal (**Figure 3E–F**), which, as the characterisation of one of them (EfTuM) suggests, could reflect a defect in protein synthesis and/or an inhibition in its exit from the ER.

The most common, by far, phenotype scored in our screen was the MG (more and smaller Golgi spots) phenotype, with 41 out of 49 hits displaying it to different degrees (**Figure 8C**). This phenotype is reminiscent of the effect of Scar/WAVE, Abi and SMSr depletion. In the first two depletions the increase in Golgi number comes from the splitting of Golgi stack pairs [8], whereas upon SMSr depletion this is accompanied by a Golgi disorganisation into tubular-vesicular clusters [9]. Distinguishing between the two possibilities in our screen hits would require the EM examination of the samples, which is not feasible for such a large number of hits. The MG phenotype appears to be nevertheless specific for the depletion of our hit genes, as depletion of 6 *bona fide* ER proteins or ER stress induction did not lead to this phenotype.

### tER-Golgi unit alterations, big cell (BC) phenotype and ER localisation

One of the surprising findings in this screen was the fact that the MG phenotype was often associated with an increased cell size (BC phenotype), as it was also the case for Scar and SMSr (**Table 1**) [8]. In addition to a larger diameter, the depleted cells also exhibited a larger nucleus (not shown), which is likely to be a consequence of the increase in cell size [56]. Indeed, the nuclear volume/size is adjusted in relationship to the cell size in order to maintain the ratio Nucleus/cytoplasm constant, and this increase is driven by an expansion of the nuclear envelope, maybe related to ER expansion). Interestingly, in 3 out of 4 gene depletions that that lead to MG phenotype, the increase in the number of Golgi spots appears to be correlated with a proportional increase in cell size, suggesting the existence of a causal relationship between the cell size enlargement and the increase in the number of Golgi. Perhaps, each tER-Golgi unit is able to support a given volume of cytoplasm and mechanisms are implemented to ensure this. However, this bi-parametric view is likely an over-simplification. Indeed, in some cases, such as the depletion of CG10664 and ER stress, this mechanism could be impaired, since the changes in the cell volume or the Golgi number are not strictly correlated, arguing in favour of a multi-parametric regulation.

Cell enlargement can take place through several mechanisms involving an increase in cytoplasmic activity in terms of amount of mRNA, protein, number of ribosomes, membrane content, nucleo-cytoplasmic transport or lipid metabolism, all processes that mostly take place in the cytoplasm. Despite our efforts to explain the observed increase by measuring changes in some of these parameters, such as increased anterograde protein transport, an increase in lipid droplets content, activation of the TOR pathway or a block in G2, we have not been able to identify a single cause for it. However, a common denominator in the functional annotation information of our hits has been their potential implication in cell metabolism (see below).

In addition, disruption in F-actin dynamics could be the reason for the BC phenotype at least in some hits, as it is the case upon Scar depletion (**Table 2**). Although gross defects in the organisation of the cortical F-actin were not observed in any of the validated hits upon visualisation with phalloidin, it is possible that these depletions may have disrupted metabolic processes or the structure of organelles, such as the ER, and this affected indirectly the organisation of actin cytoskeleton around the tER-Golgi units. Whether the MG phenotype is the result of cell enlargement, or vice versa, remains to be addressed.

Interestingly, our localisation studies suggested that there is a correlation between MG/BC phenotype upon hit depletion, and their localisation to the membrane of the early secretory pathway (ER, tER sites and Golgi complex). Indeed, out of the 6 proteins that we examined, 3 did localize to the ER, including one enriched at tER sites, and one to the Golgi, while CG10664 was targeted to mitochondria. Localisation of proteins was assessed upon C-terminal tagging with a V5 epitope. This tagging strategy could mask the putative ER retention signals leading to aberrant localisation away from the ER. However, our experience with the ER resident enzyme SMSr, which contains a typical KKxx C-terminal motif, has shown that C-terminal addition of the V5 epitope does not affect its ER retention [9], at least upon the short-term protein expression conditions used.

Even though the number of localised proteins is small and their overexpression did not show an obvious phenotype, it points to a correlation between ER localised proteins and the MG and BC phenotypes. The CPE synthase, SMSr, also belongs to this class of proteins, as it localizes to the ER and its depletion leads to MG++ and BC phenotype without affecting anterograde transport ([9] and this study). As these ER proteins are also involved in lipid metabolism, it is possible that this is a common cause for both the ER/Golgi phenotype as well as the increased cell size. As mentioned above, this was not due to ER stress, and the exact mechanism will require further research.

### Correlation of MG phenotype with specific biological processes

Gene ontology analysis using DAVID bioinformatics resources tools [19], [57] of the 49 identified hits, including the 16 that were validated and further characterised, has provided some links between the MG phenotype and certain molecular functions (**Figure 8C**).

A prominent group of hits comprises 7 proteins with annotated mitochondrial functions (CG10664, EfTuM, CG10075, CG31644, CG4769, CG4594 and Pyruvate dehydrogenase kinase). This indicates that despite the fact that the selected RNAi screen targets were enriched in ER proteins, we also picked proteins with different cellular localisations. The characterisation of the first two proteins showed that their depletion leads to a combination of reduced protein synthesis, partial inhibition of anterograde transport and a significant reduction in cell growth through a G1 arrest (**Figure 4**). Although such strong effects were not observed upon depletion of CG10075 (**Table 2**), which is predicted to act as an ubiquinol-cytochrome c chaperone in mitochondria, these results suggest that energy depletion and/or other mitochondrial dysfunction upon downregulation of these genes affect the functional organisation of the early secretory pathway.

A second functional group identified comprises 8 proteins with predicted or previously described role in protein glycosylation (CG14020, CG30036, CG7870/Alg5, O-fut1, Rumi, Ost48, l(2)not/Alg3 and Sut3). We did not test the localisation of these proteins but considering their molecular function, we expect that they will reside in the ER and Golgi membranes and some of them are predicted to be transmembrane ER proteins (Ost48 and l(2)not) (http://flybase.org/). It is, therefore, likely that they have a direct role in the organisation of the early secretory pathway either by mediating the glycosylation of key proteins involved in its structural organisation or by being themselves structural components of the tER-Golgi units [58], [59].

A third group consists of 2 out of the 8 *Drosophila* proteins that belong to the cytochrome b561/ferric reductase family (CG10165, CG10337). These proteins exhibit multiple transmembrane domains and perform an electron transfer reaction in intracellular vesicles through the mechanism of ascorbate regeneration. The typical cytochrome b561 family members are found in synaptic and other intracellular vesicles and the plasma membrane and have been implicated in neuronal functions [60], [61]. The two proteins that were identified in our screen, however, are completely uncharacterised in terms of function and their role in the organisation of the early secretory pathway needs to be addressed.

Last, several hits contain putative functional domains that are related to several aspects of cellular homeostasis and metabolism. CG10029 and some of mitochondria hits display oxidoreductase activity, and they are, therefore, their depletion is likely to affect the redox status of the cells [62]. Four proteins contain binding domains for metal ions (Zn/Fe/Cu; CG5196, CG8539, CG9430, CG17198), which normally act as activating cofactors. Such proteins function as enzymes and transcription factors implicated in a variety of metabolic processes including carbohydrate, lipid, protein and nucleic acid synthesis or degradation [63]. Finally, at least 4 proteins (CG34127, CG13284, CG6016, Pis) together with some of the mitochondrial hits are predicted to be involved in various aspects of lipid metabolism. Interestingly, Phosphatidyl-Inositol synthase (Pis) and CG6016 exhibit cytidyl diphosphate (CDP)-alcohol phosphotransferase activity and could be involved in ceramide:phosphoethanolamine (CPE) balance in *Drosophila* cells, which has also been reported for SMSr [9].

Furthermore, as mentioned above, 4 of the 6 proteins localise to the membrane of the early secretory pathway, suggesting that the observed changes in tER-Golgi unit organisation are probably due to their localisation and functional role in the early secretory pathway.

Taken together, we propose that various metabolic stresses resulting from defects in protein glycosylation, lipid and carbohydrate synthesis and catabolism, ion balance, ceramide homeostasis (SMSr), as well as mitochondria dysfunction and changes in cytoskeleton, have all effects that converge on the membranes of the early secretory pathway. This suggests a potential “link” between cell metabolism and ER/tER/Golgi structural organisation. ER stress induction also affects the organisation of the early secretory pathway, albeit in a very different way than most of our screen hits, whose depletion results in a MG phenotype.

Although thus far not understood, the novel phenotype that we describe here is not due to an inhibition in the anterograde transport, and it may not be restricted to the depletion of ER proteins. Depletion of kinases (our preliminary results) also appears to result in a similar phenotype. Whether this phenotype is limited to *Drosophila* S2 cells remains to be established.

## Materials and Methods

### Cell lines and culture

Wild-type, Delta and Fringe-GFP S2 cells were grown in Schneider's insect medium supplemented with 8% fetal bovine serum (FBS) at 26°C and have been previously described [6], [8], [64]. Fringe-GFP and Delta expression is under the regulation of a metallothionein promoter. Fringe-GFP synthesis was induced for 3 hours by adding 1 mM CuSO_4_ in the medium followed by a 2-hour chase in the presence of 100 mg/ml cycloheximide. Under these conditions, Fringe-GFP is localised almost exclusively to the Golgi stack [8].

### Antibodies

Antibodies raised against GFP, dSec23, dSec16, PDI (1D3), α-Tubulin (GTU-88), Delta (C594.9B), d120kD and dGMAP have been characterised before [6]-[8], [21], [65], [66]. Mouse monoclonal and rabbit polyclonal anti-V5 antibodies were obtained from Invitrogen and Sigma, respectively. Phalloidin-TRITC (Sigma) was used to detect F-actin. MitoTracker Red CMXRos (Invitrogen) was used to label mitochondria.

### Primary screen, imaging and data analysis

The primary ER screen was performed using dsRNAs transcribed *in vitro* from a second generation RNAi library (HD2; [67]). Complete sequence information of the dsRNAs contained in this library is available at http://www.genomernai.org.

The primary screen was performed in duplicates in 384-well plates (BD Falcon). 5 µl of each dsRNA tested (∼0.5 µg) was spotted manually at the bottom of the wells using an automated liquid dispenser (Multidrop, Thermo Labsystems) followed by the addition of 10 µl Fringe-GFP S2 cells resuspended in Schneider's Insect medium (Sigma) without serum at a concentration of 7×10^5^ cells/ml. After a short spin, the plates were incubated for 1 hour at room temperature; subsequently, 25 µl of medium containing 10% FBS, 100units/ml penicillin and 100 µg/ml streptomycin was added to each well. The plate was covered with aluminum sealing tape (Corning) and the cells were grown at 25°C for 5 days to allow for protein depletion. After the 5^th^ day, the medium was replaced with 40 µl fresh Schneider's medium containing 10% FBS and 1 mM CuSO_4_ for 3 hours followed by a 2 h chase in the presence of 100 µg/ml cycloheximide.

Each plate contained control dsRNAs in triplicate. Wells with no dsRNA and LacZ dsRNA were used as negative controls. dsRNAs targeting dSec16, Scar and Abi were used as positive controls [8], [21]. EGFP and Drosophila Inhibitor of Apoptosis 1 (DIAP1)-targeting dsRNAs [68] were used for monitoring the RNAi procedure in every experiment.

Cells were fixed in cold methanol for 7 min, rinsed twice in PBS and processed for indirect immunofluorescence. Labelling using anti-GFP (1∶300) and anti α-Tubulin (1∶2000) antibodies was performed. Goat anti-rabbit coupled to Alexa488 and goat anti-mouse coupled to Alexa568 was used as secondary antibodies (1∶200; Molecular Probes). Finally, Hoechst staining was performed to label the DNA.

Each 384-well plate was scanned on a fully automated Zeiss Axiovert 200 M widefield epifluorescence microscope. The microscope stage movement and the image acquisition was fully automated and controlled by Metamorph software. Images for each channel from 4 different positions per well were acquired using a CoolSNAP HQ (12-bit monochrom) CCD camera (Photometrics).

All captured images from Fringe-GFP channel were examined manually and the dsRNAs leading to a pattern deviating from this observed in the control cells were scored. Such deviations typically included a clear increase or decrease in the number of fluorescent puncta observed as well as increased out of focus light (haze). However, various technical difficulties and the relatively low resolution of the wide-field microscopy without applying any method to reduce out of focus light, such as deconvolution, which was used in our primary screen, precluded any reliable assignment of the depleted genes into different phenotypic classes. The dsRNAs that led to an altered Golgi pattern in both plates or only in one, in case no cells were captured in the duplicate plate, were considered as hits. If the phenotype in the two plates was different, the dsRNA effect was considered as unclear. If very few or no cells were pictured in both plates used, the dsRNA phenotype was classified as not determined (**Figure 1** **; Table S1**).

### Secondary/Confirmation screen

110 genes that were scored as positive, unclear or not determined in the primary ER screen were re-tested in a second screening set-up with the same dsRNAs that were used in the first screen (**Figure 1** **; Table S1**).

The confirmation screen was performed once in 24-well plates containing round coverslips. 70 µl of each dsRNA tested (∼7 µg) was added at the bottom of the wells followed by the addition of 180 µl Fringe-GFP S2 cells resuspended in Schneider's insect medium (Sigma) without serum at a concentration of 7×10^5^ cells/ml. After incubation for 1 hour at room temperature, 330 µl of medium containing 10% FBS, 100units/ml penicillin and 100 µg/ml streptomycin was added to each well. The cells were grown at 26°C for 5 days and then Fringe-GFP was induced with 1 mM CuSO_4_ as described for the primary screen. No dsRNA and dsRNA targeting dSec16, Scar, Abi and EGFP were used as controls.

Cells were fixed in 4% paraformaldehyde in PBS for 20 min at room temperature and processed for indirect immunofluorescence (IF) as previously described [6]. Labelling with anti-Sec16 (1∶800; [21] and anti-PDI (1D3 1∶25; [66] antibodies was performed. At the end, coverslips with attached cells were mounted in Vectashield containing Dapi.

The samples were examined under a Zeiss LSM-510 confocal microscope and the phenotypes were documented using LSM 5 Image software. Images for each channel from 2 selected fields per sample were captured.

Hits were considered all dsRNAs leading to a tER-Golgi pattern significantly deviating from this observed in the control cells. Detailed description and classification of the positive hits into groups based on the phenotype is presented in **Table S2.**

### dsRNA designing for the validation and characterisation of selected hits

The dsRNAs used for the characterisation of selected screen hits were independently designed and each probe was evaluated for its efficiency and potential off-target effects on the website http://e-rnai.dkfz.de [67]. Only probes with 100% specificity for the targeted gene were used. The primers and dsRNA sizes of each targeted gene are mentioned in **Table S3**.

### Quantification of cell diameter, number and size of tER sites and Golgi by ImageJ

As an indication for the S2 cell size, the cross-sectional diameter of equatorial cell confocal sections or 2D projections of the cells was measured using the “Measure” function in ImageJ software (Version 1.42; http://rsbweb.nih.gov/ij/). Cell diameter is expressed as percentage normalised to the diameter of mock-treated cells, which was set as 100%. Typically, each calculated average derives from at least 3 independent experiments per condition measuring a minimum of 30 cells per experiment.

The number and size of fluorescent puncta corresponding to tER sites (Sec16) and Golgi (dGMAP in Figure 7) and (Fringe-GFP in Table 1) was performed on 2D projections of confocal sections encompassing the entire cell volume, as previously described [8].

The ratio number of Golgi spot/10 µm^3^ of cell volume was estimated per cell profile with the hypothesis that the cells were spherical. About 20–30 cells were quantified per condition.

### Quantitation of cell proliferation

The number of S2 cells after 5-day depletion with various dsRNAs was counted using a haemocytometer and expressed as percentage relative to the number of mock-treated cells at the same time point, which was considered as 100%. At least 4 independent experiments were performed for each calculated average.

### Quantitation of cell cycle distribution by flow cytometry

To determine the effect on cell-cycle distribution after each RNAi depletion, living S2 cells were harvested and labeled with Vybrant DyeCycle green stain (Molecular Probes) according to the manufacturer's protocol. The DNA content was determined with a FACScalibur fluocytometer using CellQuest acquisition software (Becton Dickinson, San Jose, CA); 20,000 events were analyzed per sample per experiment. Data analysis was performed with FCS Express version 3 software (De Novo Software, Los Angeles, CA). At least 2 independent experiments were performed for each calculated average.

### Lipid droplet formation

To visualize lipid droplets, fixed S2 cells were incubated with 500 ng/ml Nile red (Sigma) in PBS for 10 min, rinsed in PBS and dH_2_O and mounted on Vectashield containing Dapi. The number of lipid droplets per equatorial confocal cell section was expressed as percentage normalised to the respective value in mock-treated cells, which was considered as 100%. Quantification is the result of at least 3 independent experiments using a minimum of 5 randomly selected fields of cells per experiment.

### Statistical analysis of quantifications

The statistical significance for all quantifications was assessed by two-tailed unpaired Student's t tests and is indicated by asterisks in the histograms and figure legends. RNAi effects deviating from mock-treated samples were considered statistically significant when p value was lower than 0.05 or 0.01 (see legend for Figures). RNAi conditions with p values between 0.05 and 0.20 were considered as an indication of a trend towards a particular phenotype, which however was not statistically significant and would require further characterisation.

### TORC1 activation

This was measured by examining the phosphorylation of S6K at Thr389 by western blot using the phospho-p70 S6 Kinase (1A5) monoclonal antibody (1∶1000; Cell Signaling). For controls, S2 cells were grown in medium containing serum supplemented or not with 3 µg/ml insulin, 10 mM rapamycin or both for 2 h before being lysed in SDS sample buffer (2×10^6^ cells/25 µl). Cells depleted of different protein hits for 5 days were directly lysed in SDS sample buffer [35].

### Cloning

To test the subcellular localisation of selected hits, the full-length coding sequences were amplified by PCR and cloned into pMT/V5-HisA,B,C vectors (Invitrogen). The expressed proteins were C-terminally tagged. The primers and restriction sites used to clone each gene are mentioned in **Table S4**.

### Transient transfections

S2 cells were transiently transfected for 2 days as previously described [7]. The expression of each tagged protein was induced for 2 h with CuSO_4_ followed by a 2 h chase sometimes in the presence of cycloheximide that was included during chase to minimize potential false localisation of transmembrane proteins in the ER.

The ER localisation was further assessed by co-transfecting the V5 tagged candidates and the mammalian ER resident enzyme PE N-methyltransferase (PEMT) tagged with GFP that also localizes to the ER in S2 cells [9].

### ER stress

S2 cells were incubated with 1 mM Thapsigargin, 10 µg/ml Tunicamycin or 5 mM DTT for up to 24 h, conditions that have been previously described to induce ER stress [38], [39]. After fixation in 4% PFA, the cells were labelled for Sec16 or dGMAP.

## Supporting Information

Table S1 **Summary of the primary RNAi screen results.**
(XLS)

Table S2 **Candidate classification after the confirmation screen based on the tER-Golgi phenotype.**
(XLS)

Table S3 **dsRNA design for further characterisation of 16 candidates.**
(XLS)

Table S4 **Primers for the cloning of 6 candidates.**
(XLS)

Figure S1 **Co-localisation of three candidates with the ER marker PEMT-GFP.** IF localization of candidates tagged with V5 in S2 cells co-expressing the ER marker PEMT-GFP. Note the extensive overlap between the two fluorescent channels (white arrows) and the thick nuclear envelope (double arrows). Scale bars: 5μm.
(TIF)

Figure S2 **The MG phenotype is due to the depletion specific ER proteins.** Visualisation of tER-Golgi units (Sec16 and Fringe-GFP, respectively) in S2 cells by confocal microscopy upon RNAi depletions of 3 *bona fide* ER proteins (ERp60, SsRbeta and CaBP1). Note that the tER-Golgi units remain largely unaffected when compared to non-depleted cells. Scale bars: 10μm.
(TIF)
