# Supplementary figures and images for: Identification of ER Proteins Involved in the Functional Organisation of the Early Secretory Pathway in Drosophila Cells by a Targeted RNAi Screen
Source: PLoS One. 2011 Feb 23;6(2):e17173. doi: 10.1371/journal.pone.0017173 (PMC3044168; doi:10.1371/journal.pone.0017173)

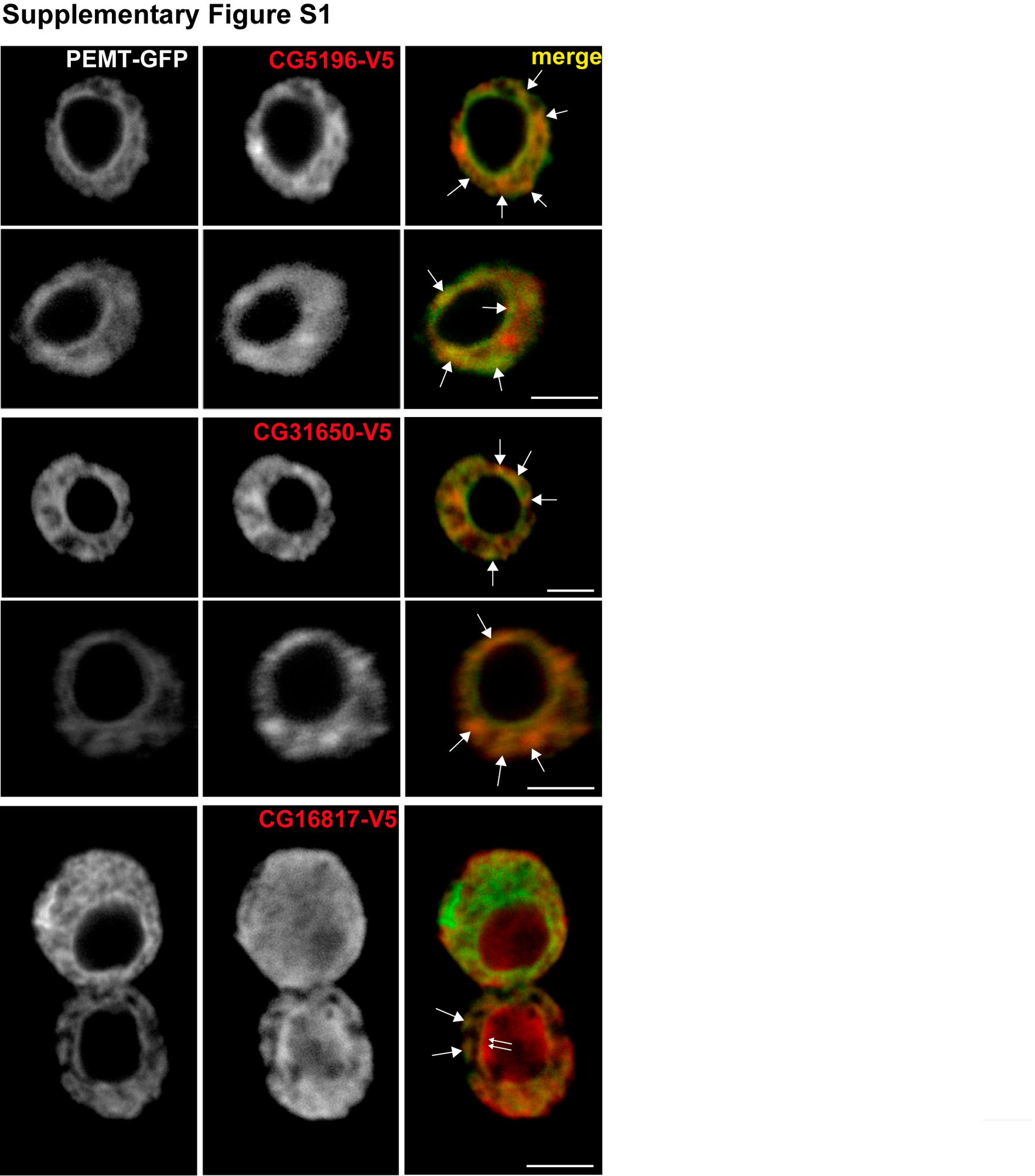

Supplement: Figure S1 — Co-localisation of three candidates with the ER marker PEMT-GFP. IF localization of candidates tagged with V5 in S2 cells co-expressing the ER marker PEMT-GFP. Note the extensive overlap between the two fluorescent channels (white arrows) and the thick nuclear envelope (double arrows). Scale bars: 5μm. (TIF) [file pone.0017173.s005.tif]

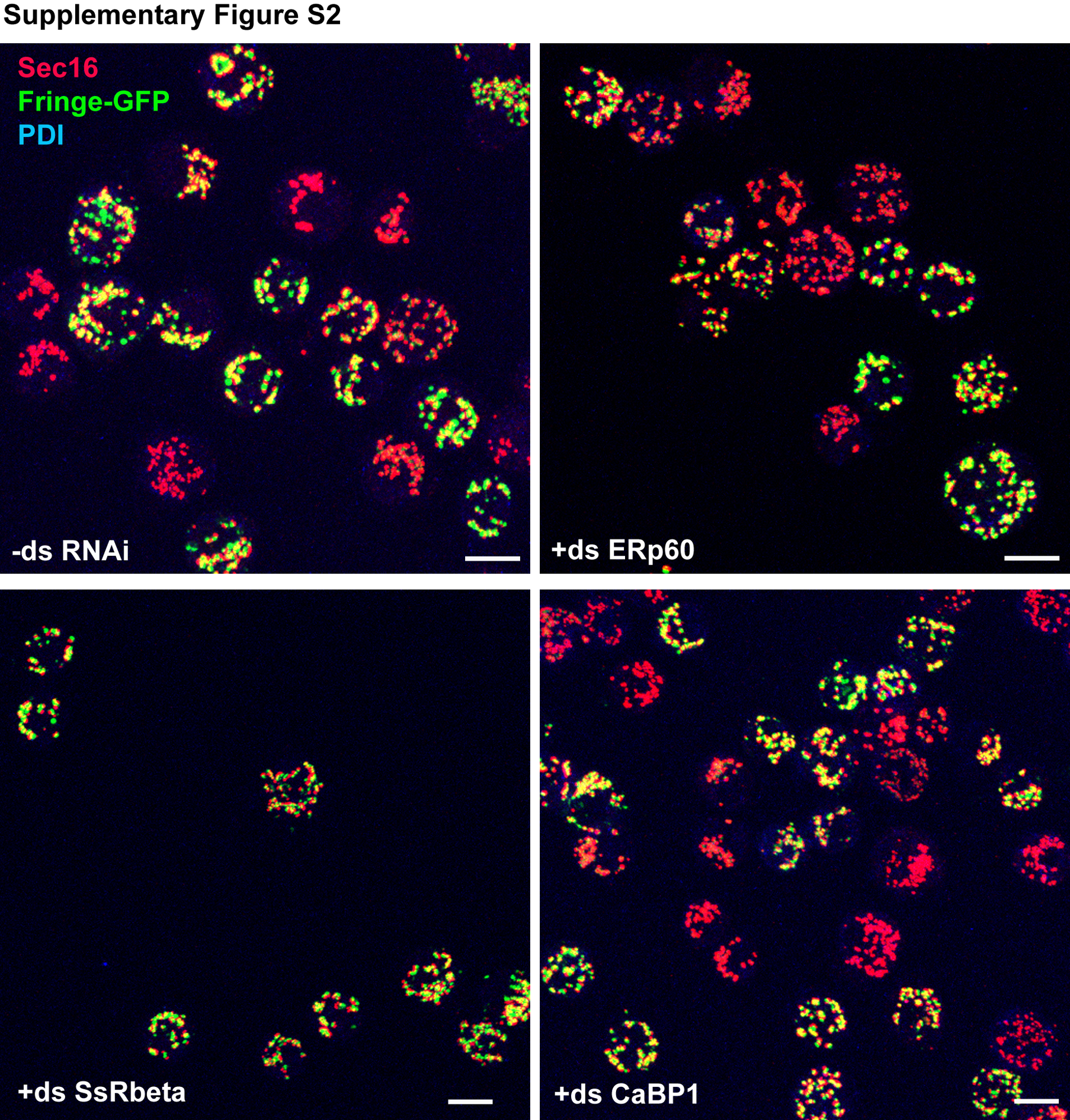

Supplement: Figure S2 — The MG phenotype is due to the depletion specific ER proteins. Visualisation of tER-Golgi units (Sec16 and Fringe-GFP, respectively) in S2 cells by confocal microscopy upon RNAi depletions of 3 bona fide ER proteins (ERp60, SsRbeta and CaBP1). Note that the tER-Golgi units remain largely unaffected when compared to non-depleted cells. Scale bars: 10μm. (TIF) [file pone.0017173.s006.tif]
